# Care transitions among oncological patients: from hospital to community

**DOI:** 10.1590/1980-220X-REEUSP-2022-0308en

**Published:** 2023-01-23

**Authors:** Caroline Donini Rodrigues, Elisiane Lorenzini, Manuel Portela Romero, Nelly Donszelmann Oelke, Vanessa Dalsasso Batista Winter, Adriane Cristina Bernat Kolankiewicz

**Affiliations:** 1 Universidade Regional do Noroeste do Estado do Rio Grande do Sul, Programa de Pós Graduação Strictu Sensu em Atenção Integral à Saúde, Ijuí, RS, Brazil.; 2 Universidade Federal de Santa Catarina, Departamento de Enfermagem, Florianópolis, SC, Brazil.; 3 Universidade de Santiago de Compostela, Faculdad de Medicina, Ciencias de la Salud, Santiago de Compostela, Spain.; 4 The University of British Columbia, School of Nursing, Vancouver, Canada.

**Keywords:** Continuity of Patient Care, Transitional Care, Neoplasms, Patient Safety, Continuidade da Assistência ao Paciente, Cuidado Transicional, Neoplasias, Segurança do Paciente, Continuidad de la Atención al Paciente, Cuidado de Transición, Neoplasias, Seguridad del Paciente

## Abstract

**Objective:**

To analyze the transition of care from the perspective of cancer patients, in a Southern Brazil hospital, correlating perspectives with sociodemographic and clinical characteristics.

**Method:**

Cross-sectional study using the Care Transitions Measure (CTM) with cancer patients undergoing clinical or surgical treatment following hospital discharge. Data collection was completed by telephone, between June and September 2019. Data analysis was performed using descriptive and inferential statistics.

**Results:**

The average CTM score was 74.1, which was considered satisfactory. The CTM factors: understanding about medications (83.3) and preparation for self-management (77.7) were deemed satisfactory; while: secured preferences (69.4) and care plan (66.1) were unsatisfactory for an effective and safe care transition. No statistically significant difference was found between sociodemographic variables and the CTM. Among the clinical variables, primary cancer and the secured preferences factor showed a significant difference (p = 0.044).

**Conclusion:**

The transition from hospital care to the community was considered satisfactory in the overall assessment.

## RESUMO

**Objetivo:**

Analisar a transição do cuidado na perspectiva de pacientes oncológicos, em um hospital do sul do Brasil, correlacionando perspectivas com características sociodemográficas e clínicas.

**Método:**

Estudo transversal utilizando o Care Transitions Measure (CTM) com pacientes oncológicos em tratamento clínico ou cirúrgico após a alta hospitalar. A coleta de dados foi realizada por telefone entre junho e setembro de 2019. A análise dos dados foi realizada por meio de estatística descritiva e inferencial.

**Resultados:**

A pontuação média do CTM de 74,1, foi considerada satisfatória. Os fatores CTM: compreensão sobre medicamentos (83,3) e preparo para autocuidado (77,7) foram satisfatórios, enquanto: preferências garantidas (69,4) e plano de cuidados (66,1) foram insatisfatórios para uma transição de cuidado efetiva e segura. Não foi encontrada diferença estatisticamente significativa entre as variáveis sociodemográficas e o CTM. Entre as variáveis clínicas, o câncer primário e o fator de preferências garantidas apresentaram diferenças significativas (p = 0,044).

**Conclusão:**

A transição da assistência hospitalar para a comunidade foi considerada satisfatória na avaliação geral.

## RESUMEN

**Objetivo:**

Analizar la transición de la atención desde la perspectiva de los pacientes con cáncer en un hospital del sur de Brasil, correlacionando las perspectivas con las características sociodemográficas y clínicas.

**Método:**

Estudio transversal utilizando el Care Transitions Measure (CTM) con pacientes oncológicos en tratamiento clínico o quirúrgico tras el alta hospitalaria. La recogida de datos se realizó por teléfono entre junio y septiembre de 2019. Los datos se analizaron mediante estadísticas descriptivas e inferenciales.

**Resultados:**

La puntuación media de la CTM, 74,1, se consideró satisfactoria. Los factores de la CTM: comprensión sobre la medicación (83,3) y preparación para el autocuidado (77,7) fueron satisfactorios, mientras que: preferencias garantizadas (69,4) y plan de cuidados (66,1) fueron insatisfactorios para una transición de cuidados eficaz y segura. No se encontraron diferencias estadísticamente significativas entre las variables sociodemográficas y las medidas de CTM. Entre las variables clínicas, el cáncer primario y el factor de preferencias garantizadas mostraron diferencias significativas (p = 0,044).

**Conclusión:**

La transición de la atención hospitalaria a la comunitaria se consideró satisfactoria en la evaluación global. (175 palavras)

## INTRODUCTION

Cancer is a complex disease, with a high incidence of morbidity and mortality, representing a worldwide public health problem^(1)^. Affected patients require continuity of care, therefore, there is a need for integration and organization of care across the health system^(2)^. Plans for health services action are essential to ensure quality and safety during care transitions.

The care transition process is defined as a set of coordinated actions for continuous patient care from the moment of admission to hospital discharge, as well as the transfer of patients between units in the same facility or between different health services^(3)^. These actions include discharge planning, health education for the patient and family, promotion of self-management of care, guidance on medications, coordination between health services, communication between teams and post-discharge monitoring^(4)^. A study carried out with hospitalized cancer patients shows satisfactory results in relation to the transition of care, with weaknesses in the care plan factor. These results were discussed with the multidisciplinary team of the investigated institution, which indicates that this aspect is justified by the failures in the educational process for the preparation of discharge, lack of care protocols for patients with cancer, lack of a reference caregiver and lack of continuity of care in primary health care^(5)^.

Care transition from hospital to the community is a critical juncture in the care journey of patients. Studies^(5,6)^. have shown that the lack of planning and preparation for discharge, the difficulty in self-management of medications, the occurrence of adverse events and the increase in readmissions are common. On the other hand, effective care transitions were associated with cost reduction in health services, hospital readmissions and improvement in patients’ quality of life^(4)^.

Studies have been published internationally that have evaluated care transition through the *Care Transitions Measure* (CTM), an instrument that assesses the quality of transitions, in cancer patients, showing averages of 51.2 to 58.8^(7)^, 66.32^(8)^ to 73.1 in the overall score. A systematic review with meta-analysis with patients with colorectal cancer points out that the implementation of a Post-discharge surveillance reduces 32% of hospital readmissions and use of standardized protocol enhanced recovery significantly reduces hospital stay by up to one day and means of hospital stay^(9)^. However, despite the fact that care transitions are an internationally explored topic, the literature is still emerging as shown by Alberta`s first Home to Hospital to Home Transitions Guideline^(10)^.

In Brazil, there have been no studies to evaluate the care transitions of cancer patients^(11)^. The results of this study will contribute to the understanding of the perceptions of patients and/or their caregivers regarding the quality-of-care transitions during hospitalization and discharge to the community. Furthermore, the results will contribute to supporting the development and implementation of safe care transitions for this group of patients, which in general has a high rate of readmissions^(12)^, evidencing the need of continuity of care. The aim of this study was to analyze care transitions from the perspective of cancer patients or their caregivers, in a hospital in Southern Brazil, and to correlate those results with sociodemographic and clinical characteristics.

## METHOD

### Design of Study

This is a cross-sectional study.

### Study Location

Was carried in an oncology unit out in a non-profit hospital located in a mid-sized city of the state of Rio Grande do Sul, Brazil. The hospital has 228 beds and average of 12,256 admissions per year. It includes a High Complexity Center for Cancer Treatment (CACON). CACON is a highly complex service that encompasses specific human and technological resources for specialized care for cancer control.

### Population

All cancer patients admitted for clinical treatment (cancer treatment or clinical complications due to cancer) or surgery, hospitalized by the Brazilian National Health System (SUS), were eligible to participate in the research. Elective admissions for chemotherapy and radiation were also included.

During the study period, 393 oncology patients were discharged from hospital; of these, 213 patients participated in our study.

### Selection Criteria

Inclusion criteria included cancer patients, aged 18 years or older, hospitalized for at least 24 hours. We included only patients with a length of stay equal to or greater than 24 hours; this was the minimum period to characterize admission to a service. Exclusion criteria included patients that had cognitive conditions that impacted their ability to answer the questionnaire. During data collection of the CTM-15, those who did not answer telephone calls after the third attempt within seven to 30 days after hospital discharge, were considered to be lost to follow-up. We excluded 8 patients and 10 patients were lost to follow-up.

### Data Collection

Data were collected between the months of June and August 2019. After consent, sociodemographic and clinical data were collected in the hospital during hospitalization, from the patients, their caregivers, or family members, as well as from the patient’s medical record. The CTM-15 instrument, validated for use in Brazil^(13)^, was completed by telephone with the patient, caregiver, or family member, within 30 days after hospital discharge. Permission for the use of the Brazilian CTM-15 was provided. 

The CTM-15 is a questionnaire designed and validated for telephone use. It measures, from the patient’s perspective, the quality of the care transition from hospital to home or between different services^(14)^. It aims to assess the quality and experience of care transitions including the transfer of appropriate information, the preparation of patients and their caregivers, support for self-management of the health condition and the inclusion of the preferences of patients and their caregivers and family members in care planning. The instrument aims to assist health professionals and managers in building strategies for efficient and safe care transitions.

The CTM-15 has four factors, divided into 15 questions: 1) preparation for self-management: the preparation of the patient and family for self-management of post-discharge health at home; 2) understanding of medications: refers to the patient and family’s understanding of the proper use of medications after hospital discharge; 3) preferences: describes the patients’ needs and preferences to be included by the care team when making treatment decisions; and 4) care plan: the existence of a care plan, including follow-up procedures to be carried out after discharge.

From the participant’s responses, a score is assigned. The instrument uses a five-point scale: I don’t know / I don’t remember / it doesn’t apply = 0; strongly disagree = 1 point; disagree = 2 points; agree = 3 points; totally agree = 4 points. To calculate averages, a formula is applied that transforms the results obtained into scores from 0 to 100^(14)^. Higher scores indicate better quality of the care transitions. Although there is no cut-off point, a score equal to or greater than 70 is considered satisfactory^(13)^.

### Data Analysis and Treatment

The data were analyzed using the program, *Statistical Package for Social Sciences* version 25.0 (SPSS Inc., Chicago/IL, USA, 2015). A level of 5% was considered to be significant. Descriptive statistics with absolute and relative distribution and measures of central tendency and variability were applied. Analysis of Variance - ANOVA (One-Way) Post-Hoc Tukey (independent groups of similar sizes) or Scheffé (independent groups of very different sizes and / or heterogeneity of variances were used in the comparison of continuous variables between two or more independent groups).

### Ethical Aspects

The research followed the recommendations of Resolution No. 466/2012 of the National Health Council (CNS). The Institutional Research Ethics Committee cleared the study by opinion 3,266,259/2019. All participants, after clarifying the objectives of the study and expressing interest, signed the Informed Consent Form (ICF) in two copies of equal content.

## RESULTS

Our study sample included 213 cancer patients. Of these, 68.1% patients lived in urban areas, 57.7% were male and 42.3% were female, and 86.2% identified as being white. Participants’ average age was 59.6 years, the majority were married or in a stable relationship (79.7%) and just over half of the sample (58.2%) had a lower education than high school. Only 13.6% were working before hospitalization.

As shown in Table 1, neoplasms of the digestive system were most common (63.4%). Clinical complications were the main reason for hospitalization (73.1%). Cancer Stages III (27.9%) and IV (38.0%) were most prevalent in participants. The presence of metastasis was confirmed in 44.2% of patients. Just over half of the cases reported having another comorbidity (52.6%). At the time of admission, 68.5% of the patients used continuous medication, 45.0% reported undergoing chemotherapy or radiotherapy and (26.9%) were admitted for surgery.

**Table 1:** Clinical characterization of cancer patients admitted to a hospital in southern Brazil – RS, Brazil, 2020. (n = 213).

Variables	Total sample (n = 213)*	
	n	%
**Staging**		
I	6	3.4
II	26	14.5
III	50	27.9
IV	68	38.0
Other*	29	16.2
**Metastasis**		
Yes	92	44.2
No	116	55.8
**Comorbidity**		
Yes	110	52.6
No	99	47.4
**Reason for hospitalization**		
Chemotherapy Treatment/Radiotherapy	95	45.0
Surgical treatment	46	21.8
Clinical complications**	70	33.2
**Use of continuous medication**		
Yes	146	68.5
No	64	30.0
Do not know	3	1.4
Tumor location***		
Respiratory System Neoplasm	14	6.6
Breast Neoplasm	14	6.6
Digestive System	135	63.4
Reproductive system	15	7.0
Leukemias/Lymphomas	9	4.2
Others****	26	12.2
**Type of cancer**		
Primary	128	60.7
Secondary	83	39.3
**Oncological treatment**		
Chemotherapy (neoadjuvant, adjuvant)	128	63.1
Surgical	42	20.7
Others*****	19	9.4
Does not perform	14	6.9

Source: Research data, 2020.

*Others: Chronic Lymphocytic Leukemias (LLC); Acute Lymphocytic Leukemia (ALL); Acute Myelocytic Leukemia (AML); Polycythemia Vera; Plasmablastic lymphoma.

**Clinical complications: pain; anemia; fatigue/tiredness/asthenia; pulmonary complications; inappetence; nausea; fever; diarrhea; oliguria; dysphagia; recurrence; jaundice; abdominal pain; convulsion; bleeding.

***Respiratory system (rhinopharynx, oropharynx, lung); Digestive system (pharynx, esophagus, gastric, colon, rectum, liver, pancreas); Reproductive system (ovary, uterus, vulva, prostate, testis); Leukemias/Lymphomas (Chronic Lymphocytic Leukemia; Acute Lymphocytic Leukemia; Acute Myelocytic Leukemia; Polycythemia Vera; Plasmablastic Lymphoma).

****Others (CNS, thyroid, urinary, renal, melanomas, soft tissues).

*****Others: radiotherapy; chemotherapy/radiation therapy; hormone therapy.

In the descriptive statistics for each of the questions of the CTM-15 scale the highest average was shown in question Q9 (3.7 ± 05) and the lowest in question Q7 (2.4 ± 07). No ceiling or floor effect were identified in the CTM-15. Only six participants (2.8%) answered all items as “strongly agree,” having the maximum score (ceiling), and no participant had the minimum score “totally disagree,” on all items of the instrument (floor).

This study demonstrated high reliability for internal consistency of the CTM-15 with Cronbach’s alpha 0.876. The general CTM-15 score ranged from 66.1 to 83.3, with an average of 74.1. When evaluating the means by factor, the highest mean was found in Understanding of medications (83.3) and the minimum in the Care Plan (66.1) (Table 2).

**Table 2:** Measures central tendency variability factors CTM-15 instrument – RS, Brazil, 2020. (n = 213).

CTM-15 estimates (n = 213)						
Questions	Average	Standard deviation	Amplitude		Median	α C
			Minimum	Maximum		
**CTM-15 GENERAL**	74.1	10.0	31.3	100.0	73.4	.876
**Factors**						
Preparation for self-management	77.7	12.1	25.0	100.0	75.0	.852
Understanding about medications	83.3	11.5	25.0	100.0	83.3	.819
Secured preferences	69.4	16.5	25.0	100.0	75.0	.932
Care plan	66.1	13.9	25.0	100.0	62.5	.696

Source: Research data, 2020. Abbreviations: α C, Alfa de Cronbach.

Table 3 shows that there was no statistically significant difference between the mean scores of the factors and the sociodemographic characteristics of the patients.

**Table 3:** Average standard deviation of CTM-15 factors according socio-demographic characteristics – RS, Brazil, 2020. (n = 213).

Variables	CTM-15 factors								
		Preparation for self-management		Understanding medications		Secured preferences		Care plan	
		Average	SD	Average	SD	Average	SD	Average	SD
**Sex**									
Male		78.4	11.8	83.2	11.0	69.9	16.5	65.6	14.2
Feminine		76.6	12.5	83.5	12.2	68.7	16.6	66.8	13.6
p value		.295		.844		.615		.523	
**Age range**									
25 to 59		78.5	13.0	84.2	11.4	70.8	16.4	67.0	15.4
60 to 69		76.9	11.6	82.9	11.0	69.8	17.5	67.0	13.0
≥70		77.0	11.0	82.1	12.2	66.4	15.6	63.5	11.9
p value		.653		.537		.281		.260	
**Marital status**									
Not married		79.0	8.5	82.9	11.3	72.0	14.9	65.3	12.7
Married or in a stable relationship		77.2	12.9	83.3	11.5	68.8	16.9	66.4	14.3
p value		.395		.846		.266		.645	
**Breed**									
White		77.3	12.4	83.2	11.6	69.5	16.7	65.8	14.3
Black or brown		79.6	10.1	84.9	11.2	71.0	17.3	66.7	12.2
p value		.353		.454		.659		.739	
**Education**									
Illiterate		77.1	14.2	80.9	18.2	76.4	17.7	68.8	18.8
Less than high school		77.1	12.8	82.8	11.1	68.9	17.0	65.8	14.3
High school		77.9	11.4	82.7	10.6	68.6	15.6	65.4	13.3
University education		80.3	8.7	88.9	10.0	70.3	15.5	67.9	10.6
p value		.731		.100		.490		.814	
**Worked before hospitalization**									
Yes		76.7	11.1	84.3	10.4	71.3	14.5	64.7	15.0
No		77.8	12.3	83.1	11.7	69.1	16.8	66.3	13.8
p value		.658		.598		.514		.545	
**Place of residence**									
Urban area		78.2	11.7	83.3	11.2	68.7	16.5	66.4	13.3
Countryside		76.4	12.9	83.2	12.1	70.9	16.6	65.5	15.3
p value		.316		.970		.366		.684	

Source: Research data, 2020. Abbreviations: SD, Standard Deviation.

In the CTM-15 factor of Assured preferences, patients with primary cancer (71.4 ± 16.9) had a higher mean, with a statistically significant difference (p = 0.044) when compared to the mean of patients with secondary cancer (66.8 ± 15.1). Other clinical variables did not show statistical differences on other CTM-15 factors (Table 4).

**Table 4:** Mean standard deviation of CTM-15 factors according to clinical characteristics – RS, Brazil, 2020. (n = 213).

Variables	CTM-15 factors								
		Preparation for self-mana gement		Understanding medications		Secured preferences		Care plan	
		Average	SD	Average	SD	Average	SD	Average	SD
**Staging**									
I		81.3	15.3	86.8	13.5	72.2	17.2	75.0	13.7
II		80.0	10.7	84.6	11.8	69.1	16.1	66.1	13.9
III		76.4	15.7	82.7	13.4	70.5	16.8	62.1	16.4
IV		77.0	10.7	83.4	9.6	68.7	16.2	67.9	11.6
Other*		80.2	9.6	84.7	11.2	67.9	20.1	69.1	16.2
p value		.513		.876		.948		.076	
**Metastasis**									
Yes		77.0	9.5	83.6	9.5	67.8	16.3	66.6	10.5
No		78.6	13.6	83.3	12.4	70.9	16.8	66.2	15.9
p value		.338		.824		.177		.866	
**Comorbidities**									
Yes		76.9	11.2	83.9	11.3	68.2	16.4	65.2	13.0
No		78.4	13.0	82.6	11.8	70.6	16.6	66.8	14.8
p value		.383		.419		.287		.416	
**Reason for Hospitalization**									
Chemotherapy Treatment/Radiotherapy		77.2	12.6	83.1	11.3	68.8	16.1	65.6	14.0
Surgery		78.4	12.7	80.8	11.9	73.2	16.8	67.9	14.3
Clinical complications**		77.6	11.3	85.0	11.5	67.2	16.6	66.1	13.6
p value		.867		.156		.149		.640	
**Use of continuous medication**									
Yes		77.4	12.2	84.0	12.0	69.2	16.5	65.7	14.0
No		78.3	12.3	81.5	10.3	69.9	17.1	67.3	14.2
Do not know		75.6	1.1	87.2	4.6	67.4	8.4	63.5	1.7
p value		.857		.311		.947		.704	
Tumor location***									
Respiratory system		78.7	9.4	83.3	9.7	63.8	14.9	64.5	11.9
Breast		76.0	17.3	81.8	18.1	73.4	14.6	68.1	16.0
Digestive system		77.8	12.1	83.7	11.4	69.8	16.8	66.0	14.7
Reproductive system		79.3	11.0	82.7	10.7	71.8	16.6	65.2	10.8
Leukemia		82.2	12.2	86.0	12.7	68.7	19.4	72.6	16.1
Others****		74.8	10.8	81.4	8.9	67.1	16.4	65.0	10.6
p value		.658		.894		.657		.762	
**Type of cancer**									
Primary		78.9	11.9	83.6	11.1	71.4	16.9	66.1	14.9
Secondary		76.2	12.0	82.8	12.2	66.8	15.1	66.1	12.5
p value		.116		.649		.044		.989	
**Current cancer treatment**									
Chemotherapy (neoadjuvant/adjuvant)		77.2	11.8	84.0	11.0	67.4	17.3	65.7	13.8
Surgical		77.5	10.8	82.0	10.1	71.8	17.3	66.4	11.2
Others*****		77.9	16.4	81.4	15.2	71.8	12.1	68.9	17.2
Does not perform		77.1	13.3	80.8	14.2	73.5	13.8	63.8	17.3
p value		.995		.567		.271		.746	

Source: Research data, 2020.

*Other: Chronic Lymphocytic Leukemias (LLC); Acute Lymphocytic Leukemia (ALL); Acute Myelocytic Leukemia (AML); Polycythemia Vera; Plasmablastic lymphoma.

**Clinical complications: pain; thrombocytopenia; anemia; fatigue/tiredness/asthenia; pulmonary complications; inappetence; nausea; fever; diarrhea; oliguria; dysphagia; cancer recurrence; jaundice; abdominal pain; convulsion; bleeding.

***Respiratory system (rhinopharynx, oropharynx, lung); Digestive system (pharynx, esophagus, gastric, colon, rectum, liver, pancreas); Reproductive system (ovary, uterus, vulva, prostate, testis); Leukemias/Lymphomas (Chronic Lymphocytic Leukemia; Acute Lymphocytic Leukemia; Acute Myelocytic Leukemia; Polycythemia Vera; Plasmablastic Lymphoma).

****Others (CNS, thyroid, urinary, renal, melanomas, soft tissues).

*****Others: Radiotherapy, Chemotherapy plus Radiotherapy, Hormone Therapy.

Abbreviations: SD, Standard Deviation.

## DISCUSSION

The results of this investigation made it possible to assess the quality-of-care transitions from the perspective of cancer patients discharged from hospital. Sociodemographic and clinical characteristics were also identified and compared to CTM-15 factor scores. Our key findings indicated poor scores for the care plan and assured preferences CTM factors highlighting the need for improvement. The overall average of the CTM was satisfactory. Similar values were obtained in others studies conducted in Brazil^(6,13)^ indicating moderate quality in care transitions at the time of hospital discharge.

Our results concur with international data, as pointed out by the first Alberta´s Home to Hospital to Home Transitions Guideline, which indicates that near 30 per cent of patients in Alberta experience a gap in care during their transition from hospital to home. To address this gap, the provincial government launched the guideline targeting a standard approach to transitions, which enables the understanding of care transition processes from all involved in a transition. Above all, improvement in patient outcomes, experience and satisfaction are expected. Also, the approach will bring provider satisfaction and enable a collaborative team attitude to providing patient-centered care^(10)^.

In our study, among factors of the CTM, preparation for self-management obtained a satisfactory average. Self-management of health conditions is influenced by the understanding or not of the information provided by health professionals, as well as the attention given to answering the patient or family’s questions. This is an important component of care transitions that requires commitment from both, professionals and patient/family to avoid insecurity and uncertainties regarding the necessary care after discharge^(4)^.

The literature also highlights that the patient’s place of hospitalization is associated with the preparation for self-management of health after hospital discharge. A previous study^(13)^ showed that patients hospitalized in clinical inpatient units evaluated better this factor, due to the availability of professionals and greater opportunities to prepare the patient for discharge. Furthermore, professionals see discharge planning as part of their work. However, for patients that remain several days in emergency department due to lack of hospital beds, aspects of care transitions become more complex, and a lower score can be attributed to insufficient time of health care providers to prepare the patient and family to be discharged. Overcrowding and excessive work overload also impact health care providers’ time. Cancer patients in our study were all from clinical and surgical inpatient units, which may explain the higher CTM-15 score regarding their perception of feeling better prepared to manage their health condition.

Aspects related to the CTM-15 factor, understanding of medications, were also evaluated in another study with patients with chronic disease^(13)^, finding to have the lowest average score of all CTM-15 factors. In contrast, the current study demonstrated that this factor was positively evaluated by cancer patients, with the highest average found among all factors of the CTM-15. Studies have identified that the implementation of care protocols for medications based on scientific evidence that aim to avoid the occurrence of adverse events and maintain patient safety, are indispensable in all health institutions, as they improve care, organize health services, with the establishment of flows, and are imperative in improving the quality of care provided to the patient. In addition, having a routine review of medications and care plans by the interprofessional team helps to identify issues and the need for improvements in education for the patient and family^(15)^. Thus, information about medications, their use, dosage, and side effects are paramount for patients^(16)^.

The factors of the CTM-15, care plan and assured preferences, were assessed as unsatisfactory by cancer patients. These results require strategies to overcome this gap in this study location. Similar results have also been reported in other surveys^(13)^.

Ensuring preferences in relation to the care process of cancer patients is paramount when making care decisions post-discharge. Considering these preferences is necessary to plan actions to provide patient-centered care. Therefore, including these individuals in the preparation of a care plan, where individual preferences and needs are taken into account, tends to minimize fragmented care and optimize discharge planning^(13,17)^.

Our study showed that the care plan was not prioritized in the care of cancer patients. However, a well-designed and individualized care plan is needed to provide continuity of care, in addition to enabling an adequate care transition between the different health services in which this patient was receiving care. However, gaps and disarticulation in the health system in Brazil causes the lack of referrals and monitoring of health concerns and treatment^(6)^. Another concurrent study pointed out that there are weaknesses in communication between service professionals, which weakens the care plan for cancer patients^(18)^.

Ideally, advanced care planning, which can start at the time of hospitalization or even as an outpatient, guarantees an individualized care plan that includes patient/family preferences, instructions on medications, social support for access to health services, symptoms, warning signs and clinical monitoring. This makes the objectives of care clear and precise between the patient and health services, and between health care providers^(19)^. Therefore, it is imperative that health institutions aim to promote adequate and safe care transitions for their patients. Ideally, through strategies aimed at health education and self-care planning, involving patients and health professionals in developing an individualized care plan that considers medication reconciliation and treatment adherence can result in reduced hospital readmissions^(4)^.

The profile of the cancer patient identified in our research is in agreement with findings from other Brazilian studies, with a predominance of males, white people, with low education, over 50 years of age and stage III cancer diagnosis^(20,21)^. Also, we identified a higher prevalence of patients with neoplasms of the digestive system (64%). Malignant neoplasms of the digestive tract occur frequently in the population and are practically incurable once spread throughout the body, since the late development of symptoms is a hallmark of this type of cancer. This is reflected in diagnoses in more advanced stages^(21)^, thus requiring hospitalization for treatment. Still, the sociodemographic variables did not present statistically significant association with the CTM-15 score, converging with a study carried out in Israel with cancer patients, which also found no significant difference between groups^(7)^.

Patients with primary cancer showed a statistically significant difference in the assured preferences factor of the CTM-15 (p = 0.044), when compared to the average of patients with secondary cancer. The other clinical variables did not show statistical differences related to the CTM-15 factors. Another study carried out using CTM-15 in cancer patients that compared the groups also found no statistical differences^(17)^. Due to the lack of large studies with oncology patients and CTM measures, it is not possible to point out possible or potentially associated factors that could be addressed to explore possibilities for strengthening care transitions from hospital to community in this group of patients.

Cancer is a chronic condition, with psychological and physical changes, requiring complex and long-term care with the participation of multidisciplinary health professionals^(20)^. Added to the precedent, oncology patients require access to the necessary drugs and equipment, and it is necessary that they and their family, are fully attended to, through the provision of health education and the involvement of the patient and family in the preparation for self-management in health and social support. Continuity of care is essential and requires connection with all points of the health care system to facilitate effective and comprehensive care aimed at treating health problems.

Care transitions are considered a complex process that requires coordination and communication between the people involved, using clinical protocols, in addition to the organization and integration across the entire health care system. For these reasons, effective care transitions still challenge the integration and continuity of care for all patients, but particularly for cancer patients, as found in this study. 

### Clinical Implications

Our key findings indicated poor scores for the CTM factors of care plan and assured preferences, highlighting the need for improvement. Further studies are needed to understand the reasons why the secured preferences factor and the care plan have lower scores, which in practice make it difficult to provide or even prevent patient-centered care.

### Study Limitations

As limitations of the study, we highlight the cross-sectional design, in addition to the inclusion of cancer patients only, admitted to a single hospital. Still, the results are important, as this is the first study conducted in Brazil with this profile of patients. From the analysis of these results, we suggest carrying out further research, applying mixed methods to try to understand in depth the reasons why the patients, preferences and the care plan did not receive appropriate attention from professionals, which hinders or may even prevent patient-centered care. Furthermore, qualitative perspectives from patients and families could also be beneficial for this purpose.

## CONCLUSION

Care transitions, assessed using the CTM-15, were considered satisfactory by cancer patients admitted to clinical and surgical units of a hospital in Southern Brazil. The factors of the CTM-15 preparation for self-management and understanding of medications showed positive results. The factors of patient preferences and care plan showed lower averages, considered insufficient for an effective and safe care transition. The results contribute to supporting the development of strategies to improve care transitions among the different sections in the institution, as well as among other health services in the health care system.
